# A scoping review of social‐behaviour change techniques applied in complementary feeding interventions

**DOI:** 10.1111/mcn.12882

**Published:** 2019-09-09

**Authors:** Aimee Webb Girard, Emma Waugh, Sarah Sawyer, Lenette Golding, Usha Ramakrishnan

**Affiliations:** ^1^ Doctoral Program in Nutrition and Health Sciences, Laney Graduate School Emory University Atlanta Georgia; ^2^ Hubert Department of Global Health Emory University Atlanta Georgia; ^3^ Save the Children Washington DC USA

**Keywords:** complementary feeding, LMIC, behaviour change, review

## Abstract

Education and other strategies to promote optimal complementary feeding can significantly improve practices, but little is known about the specific techniques successful interventions use to achieve behaviour change. We reviewed the literature for complementary feeding interventions in low‐/middle‐income countries (LMIC) published since 2000. We systematically applied a validated taxonomy mapping process to code specific behaviour change techniques (BCTs) used in each intervention; effectiveness ratios for each BCT were estimated. Sixty‐four interventions met inclusion criteria, were abstracted, BCTs identified, and coded. Dietary diversity was the most commonly assessed component of complementary feeding, and interpersonal communication, either individually or in groups, was the most commonly used delivery platform. Of the 93 BCTs available for mapping, the 64 interventions included in this review applied a total of 28 BCTs. Interventions used a median of six techniques (max = 13; min = 2). All interventions used “instruction on how to perform the behaviour.” Other commonly applied BCTs included “use of a credible source” (*n* = 46), “demonstration of the behaviour” (*n* = 35), and “providing information about health consequences” (*n* = 30). Forty‐three interventions reported strategies to shift the physical or social environment. Among BCTs used in >20 interventions, five had effectiveness ratios >0.8: “provision of/enabling social support”; “providing information about health consequences”; “demonstration of the behaviour”; and “adding objects to the environment” namely, food, supplements, or agricultural inputs. The limited reporting of theory‐based BCTs in complementary feeding interventions may impede efforts to improve and scale effective programs and reduce the global burden of malnutrition.

**Key Messages**

- Behaviour change interventions to improve complementary feeding practices in low‐/middle‐income countries (LMIC) use a limited set of behaviour change techniques to effect change and depend heavily on education focused change techniques.
- Behaviour change techniques that offer opportunities for social support, create enabling physical environments, and improve self‐efficacy through self‐monitoring, goal setting, rehearsal, and problem solving hold promise and should be further evaluated.
- Detailed and publically available dissemination of intervention details, especially regarding the design approach, change techniques and implementation of complementary feeding behaviour change interventions in both high and LMIC is needed to support information sharing, identification, replication, and scaling of effective approaches.
- Implementation science research is needed to identify, develop, and evaluate optimized packages of behaviour change techniques for complementary feeding interventions.

## 1 INTRODUCTION

Breastmilk is recognized as the optimal food for infants, but beginning around 6 months of age, breastfed infants cannot meet their nutrient needs from human milk alone. Therefore, appropriate complementary feeding practices are essential to optimize child growth and development especially from 6 to 24 months of age when children are at high risk of undernutrition (Dewey, Lutter, Martines, & Daelmans, 2001). Appropriate complementary feeding encompasses the provision of (a) age‐appropriate amounts of hygienically prepared food each day in terms of meals per day and amount of food served per meal; (b) foods of adequate thickness to ensure energy density; and (c) a sufficiently diverse diet, consisting of four or more food groups daily, to ensure micronutrient requirements are met (Dewey, 2001; WHO, 2008a; WHO, 2008b). Despite considerable efforts, progress to improve complementary feeding practices in low‐/middle‐income countries (LMIC) has been slow. For too many infants, complementary foods are of inadequate nutritional quality, introduced too early or too late, or are provided in insufficient quantity or frequency. From 1990 to 2010, less than one third of children aged 6–24 months received adequate dietary diversity and only about half received a sufficient number of meals each day (Lutter et al., 2011); 20% were fed a diet that met minimum meal adequacy requirements. These numbers remained relatively unchanged from 2010 to 2016 (J. M. White, Begin, Kumapley, Murray, & Krasevec, 2017).

Initiation and maintenance of sustained behaviour change is foundational to improving complementary feeding practices and child nutrition. Recent systematic reviews of behaviour change programs designed to improve complementary feeding practices found that although practices can be changed for the better, the degree of improvement and impacts on child nutrition are often small (Dewey & Adu‐Afarwuah, 2008; Graziose, Downs, O'Brien, & Fanzo, 2018; Heidkamp, 2017; Lamstein et al., 2014; Lassi, Das, Zahid, Imdad, & Bhutta, 2013). Those achieving significant changes used formative research to identify critical barriers and facilitators of optimal practice of behaviour and applied these findings to shape processes and strategies to address behavioural change at the individual or societal level. Effective interventions also clearly delineated the impact pathway of the intervention, specifying the steps from behaviour change activities to nutritional impacts. Of concern, however, is the relatively limited use or explicit specification of individual behaviour change theory in the design and implementation of complementary feeding interventions (Briscoe & Aboud, 2012; Pelto, Martin, van Liere, & Fabrizio, 2016). Use of theory facilitates rigorous intervention design, allowing programmers to explicate the proposed mechanisms through which an intervention is hypothesized to change behaviour. Use of theory also enables comparison of interventions and aids intervention replication (Breuer, Lee, De Silva, & Lund, 2016; Mayne & Johnson, 2015).

Beyond these requirements is the need to replicate interventions to both verify impacts and enable scale up. However, replication proves challenging for complementary feeding interventions. Intervention design frameworks for behaviour change and implementation details that allow bridging of activities to specific behaviour change are rarely reported in manuscripts and reports. Recently, the Behaviour Change Wheel framework was developed to better support intervention designers, implementers, and evaluators (Michie, Atkins, & West, 2014; Michie, van Stralen, & West, 2011). The Behaviour Change Wheel draws on the Capabilities, Opportunities, and Motivations framework for behaviour change (COM‐B). The COM‐B model specifies three behavioural constructs—capability, opportunity, and motivation (COM)—that interact for initiation and maintenance of behaviour change (‐B). Each component of the model (C, O, and M) can be further subdivided into 15 theoretical domains using the Theoretical Domains Framework (TDF; Cane, O'Connor, & Michie, 2012; French et al., 2012). Nine intervention functions, identified by literature review and expert consensus, encompass the vast majority of possible intervention categories that map to the COM‐B and subsequent TDF to promote behaviour change. From these broad categories, framework developers applied and iterative DELPHI approach to derive and validate a taxonomy of 93 hierarchically organized behaviour change techniques (BCTs; Abraham & Michie, 2008; Michie et al., 2013; Michie et al., 2015). BCTs, often referred to as the “active ingredients” of a behaviour change intervention, are specific, irreducible actions that can be observed and replicated. In the Behaviour Change Wheel framework, BCTs can be linked to intervention functions and a TDF and, when taken together, systematically outline how behaviour change programming is meant to affect behaviour change. Identification of BCTs allows researchers to isolate the effective components within an intervention for purposes of replication or comparison across behaviour change interventions. Specific BCTs that are part of an intervention can be identified through coding components according to a taxonomy and mapped for each intervention. Mapping then allows researchers to identify, categorize, and synthesize BCTs across interventions.

Because behaviours and the interventions designed to change them are often complex, identifying BCTs not only allows researchers to identify the component of an intervention that has an effect on behaviour but also supplies a standard for comparing causal mechanisms across interventions.

Identification and mapping of BCTs has been applied to numerous health care and population level behaviour change interventions (Michie et al., 2014), including diet‐related behaviour change interventions for obesity reduction in North America and Europe (Martin, Chater, & Lorencatto, 2013; Tate et al., 2016). However, this approach has had limited application to child nutrition in low‐/middle‐income (LMIC) settings (Aboud & Singla, 2012). Such taxonomy mapping would not only support the identification of effective techniques for adoption and scale up and but also highlight less utilized techniques that may hold promise but require further research. For purposes of this scoping review, we applied the BCT taxonomy‐v1 (BCTTv1; Michie & Abraham, 2008; Michie et al., 2013) to identify and map BCTs reported in complementary feeding interventions in LMIC.

## 2 METHODS

### 2.1 Search strategy

To identify commonly reported BCTs for complementary feeding interventions, we systematically reviewed Pubmed, PsychInfo, and Web of Science databases and hand searched relevant previous reviews. Systematic reviews of complementary feeding interventions have been previously conducted to evaluate intervention effectiveness and were used as launching off points for snowball identification of potentially relevant publications. Two reviews in particular (Graziose et al., 2018; Lamstein et al., 2014) examined the impacts of complementary feeding behaviour change interventions on diets, growth, and anaemia of children <36 months residing in LMIC. Publications included in these reviews were published in the peer‐reviewed and grey literature between 2000 and 2016. The included and excluded studies identified from these reviews were screened by the first author and two research assistants for inclusion in taxonomic mapping per the eligibility criteria below. To supplement this list with more recent research, the first author replicated the search strategy of Graziose et al., to identify additional eligible studies published between January 2016 and December 2017. Searches were conducted in PubMed, Embase, and PsychInfo and included the following search terms: “complementary feeding” OR “child feeding” OR “infant feeding” AND “behavior change” or “behavior change communication” OR “nutrition education” OR “social marketing” OR “social and behavior change” AND “behaviours” or “practices” OR “stunting” OR “growth” OR “underweight” OR “anemia” OR “wasting.” A total of 396 titles were returned from this search. The first author reviewed titles for relevance and then further screened abstracts. Final eligibility confirmation involved full‐text review. A total of 15 full‐text articles were retained from this search for abstraction. To supplement peer‐reviewed and grey literature searches, we also identified documents through searches of program‐specific websites, namely, Alive & Thrive [Link](Bangladesh, Ethiopia, India, and Vietnam), Windows of Opportunity (CARE, multicountry), Nutrition at the Center (CARE, multicountry), Enhanced Homestead Food Production (Helen Keller International, multicountry), Shouhardo II (CARE, Bangladesh), Empowering New Generations to Improve Nutrition and Economic Opportunities (ENGINE, Save the Children, Ethiopia), Micronutrient and Health (MICAH, World Vision, multicountry), and Realigning Agriculture to Improve Nutrition (RAIN, Concern Worldwide, Zambia). Of the five multicountry programs identified, documentation suggested that programs were tailored to the country contexts and activities differed accordingly. However, detailed reports of country‐specific activities were only found for Alive & Thrive and a limited number of Helen Keller International Homestead Food Production programs (Nepal, Cambodia, and Burkina Faso), thus limiting country‐specific mapping of BCTs for several programs.

### 2.2 Inclusion/exclusion criteria

Studies and programs were eligible for inclusion if they aimed to shift complementary feeding behaviours (diet diversity, meal frequency, food thickness, consumption of animal source foods, meal volume, consumption of targeted food groups, and responsive feeding) among children 6–24 months using social and behaviour change (SBC) strategies, including direct nutrition education. Nutrition‐sensitive strategies (agriculture and women's empowerment) and strategies that provided supplements or special foods/crops were also eligible if they included an explicit nutrition education or SBC component and aimed to improve complementary feeding practices in the target population. To be eligible, exposure to nutrition education/SBC activities needed to occur at least monthly and for a duration of at least 3 months. Only studies/programs in LMIC published in English between January 2000 and December 2017 were included. Because the review focused on the type of intervention techniques used, we did not exclude studies based on evaluation design, though evaluation design was taken into account when examining BCTS in relation to program effectiveness. Due to the detailed nature of the information needed about the intervention we excluded conference abstracts for which no full‐text article could be located.

### 2.3 Data abstraction, analysis, and summary measures

Data from all full‐text studies and reports that met inclusion/exclusion criteria were abstracted into a standardized form. We abstracted key variables with regard to the study identifiers and context, study design and limitations, intervention details, evaluation strategy, and outcomes evaluated. Additionally, information on the theory of change or program impact pathway, intervention design approach, behavioural theory, and whether the intervention was informed through formative research was abstracted if provided. Program activities were detailed by targeted behaviours, key messages, activity format (Lamstein et al., 2014), activity location, duration, and intensity. Outcomes were categorized as follows: knowledge, attitudes, and beliefs; antecedent outcomes (self‐efficacy, motivation, intentions); behavioural/practice outcomes; and health outcomes. Data were abstracted at the programmatic level; if multiple articles described the same program, their information was compiled and reported as a single program. Multicountry programs were abstracted by country of implementation when sufficient country‐specific information was provided (e.g., Alive & Thrive programs in Ethiopia, Bangladesh, and Vietnam were abstracted as separate programs).

### 2.4 BCT coding and taxonomy mapping

The primary objective of this review was to understand *how* complementary feeding interventions aim to change behaviour, irrespective of whether they were successful. This approach differs from previous reviews focusing on *whether* complementary feeding interventions changed behaviour. To achieve this, we applied the taxonomy of BCTs (Michie et al., 2015; West, van Stralen Maartje, & Michie, 2011) to identify and code BCTs used in the included interventions (Figure S1). The developers of the BCT taxonomy developed a coding manual and an online program that trains users in the mapping of BCTs onto interventions (http://www.bct-taxonomy.com/). Two team members completed online training on BCT taxonomy coding. Abstraction began with the trained team members abstracting four articles simultaneously and reviewing each article together with the principal investigator. Team members then independently abstracted study information and compared abstractions for discrepancies. Discrepancies were resolved in collaboration with the first author. BCT coding was completed by both graduate research assistants (GRAs) working together to maximize consistency. Abstraction and coding were reviewed weekly with the first author.

Disagreements in coding were discussed and resolved with the first author. Of note, in coding environmental opportunity‐related BCTs (i.e., 12.1 restructuring the physical environment and 12.5 adding objects to the environment), we coded food, supplements, rations, and other items that could be immediately consumed by the child with minimal preparation as 12.5. Additionally, we coded agriculture and kitchen inputs used to improving the home food environment or the ability of the household to prepare optimal foods as both 12.1 and 12.5 because they were objects added to the environment that served to restructure the environment.

### 2.5 Assessing BCTs in relation to effectiveness

To assess BCTs in relation to effectiveness, we further examined interventions that evaluated outcomes related to complementary feeding practices. We limited this assessment to quasi‐experimental or randomized controlled studies and that included at least baseline and endline data. We then divided trials into effective versus noneffective for four WHO‐recommended complementary feeding outcomes—diet diversity, minimum acceptable diet, feeding frequency, and thick porridges—and macronutrient and/or micronutrient intake data. We did not include timely introduction of complementary foods at 6 months in estimation of effectiveness ratios because this practice more closely links with exclusive breastfeeding. The environmental, social, and behavioural determinants of delaying the introduction of complementary foods to 6 months maintaining optimal exclusive breastfeeding (EBF) likely differ from those of providing optimal complementary feeding; thus, BCTs for EBF and optimal complementary feeding likely differ. The criteria for assessing effectiveness in improving practices was defined as a significant difference (*P* < .05) in these behaviours at follow‐up between groups. We developed a percentage effectiveness “ratio,” which represented the ratio of the number of times each BCT was a component of an intervention in an effective trial divided by the number of times they were a component of all trials, including noneffective trials (Martin et al., 2013).

We acknowledge that a primary limitation of the BCT taxonomy approach to coding and estimation of effectiveness ratios relies on the level of detail authors provide in manuscripts and publically available documents. We used identified manuscripts and reports as resources for additional snowball searching to locate additional details on interventions and programs; however, it was beyond the team's capacity to contact implementers for curricula, implementation details, and intervention activities not made publically available. Given this limitation, it is possible that BCTs are underreported potentially biasing effectiveness ratio estimates.

## 3 RESULTS

### 3.1 Description of included interventions

**Figure 1:**
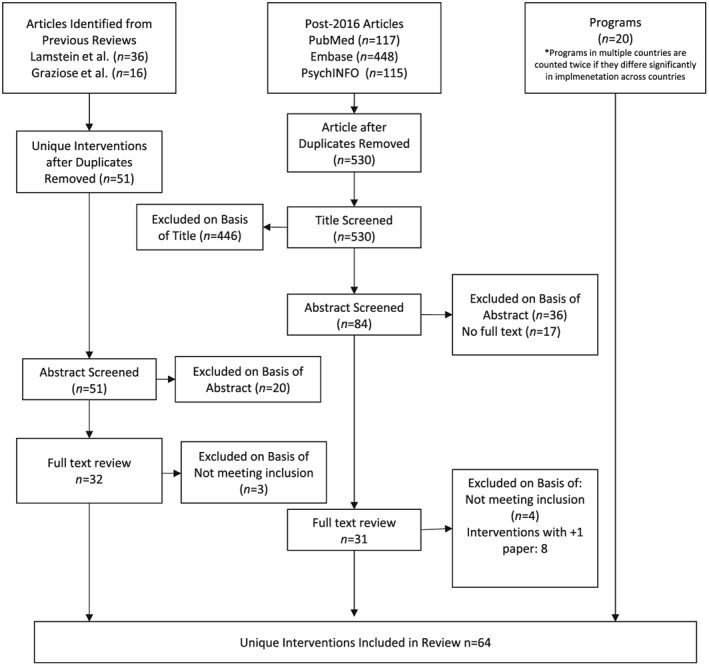
Flow chart depicting search strategy and article inclusion

A total of 64 unique social behaviour change interventions targeting complementary feeding articles were identified from previous reviews, a search of articles published since July 2016 and grey literature searches for program reports (Figure 1).

Twenty‐two interventions occurred in sub‐Saharan Africa (Aidam et al., n.d.; Aubel, Touré, & Diagne, 2004; Alive and Thrive Ethiopia, 2018; Berti, Mildon, Siekmans, Main, & Macdonald, 2010; Bruyeron, Denizeau, Berger, & Trèche, 2010; Chiutsi‐Phiri et al., 2017; Crossman et al., 2017; Fernald et al., 2016; Gelli et al., 2017; Gelli et al., 2017; Guyon et al., 2009; Harris et al., 2016; Kim et al., 2015; Kimani‐Murage et al., 2013; Kulwa et al., 2014; Leroy, Olney, & Ruel, 2016; Mukuria, Martin, Egondi, Bingham, & Thuita, 2016; Mulualem, Henry, Berhanu, & Whiting, 2016; Nikiema et al., 2017; Olney, Pedehombga, Ruel, & Dillon, 2015; Parvanta, Thomas, & Zaman, 2007; Sarrassat et al., 2015; Save the Children, 2016; Webb Girard et al., 2017; Alive and Thrive, 2016) and 11 in Bangladesh (Aboud & Akhter, 2011; Aboud, Moore, & Akhter, 2008; Aboud, Shafique, & Akhter, 2009; Frongillo et al., 2017; Hoddinott, Ahmed, Ahmed, & Roy, 2017a; Kimmons et al., 2004; Owais et al., 2017; Parvanta et al., 2007; Roy et al., 2005; Roy et al., 2007; TANGO International, 2015). Of the remaining, nine occurred in India (Alive & Thrive India, n.d.; Bhandari et al., 2001; Bhandari et al., 2004; Dongre, Deshmukh, & Garg, 2011; Kadiyala, Morgan, Cyriac, Margolies, & Roopnaraine, 2016; Kilaru, Griffiths, Ganapathy, & Ghosh, 2005; Palwala et al., 2009; Singh et al., 2017; Vazir et al., 2013) and eight Central/South American or Mexico (Bonvecchio, Pelto, Escalante, & Monterrubio, 2007; de Oliveira, Giugliani, Santo, & Nunes, 2012; Iannotti et al., 2017; Jensen, Frongillo, Leroy, & Blake, 2016; Leroy et al., 2008; Monterrosa et al., 2013; Penny et al., 2005; Santos et al., 2001). Five were implemented in China (Guldan et al., 2000; Li et al., 2007; Sun et al., 2011; J. Zhang, Shi, Chen, Wang, & Wang, 2013; Y. Zhang et al., 2016); three in Vietnam (Bruyeron et al., 2010; Dickey et al., 2002; Nguyen et al., 2014); two each in Cambodia (Olney, Talukder, Iannotti, Ruel, & Quinn, 2009; Reinbott et al., 2016) and Nepal (Cunningham et al., 2017; Osei et al., 2017); and one each in Indonesia (S. White et al., 2016), Pakistan (Zaman, Ashraf, & Martines, 2008), and Egypt (Brasington et al., 2016). Target reach ranged from less than 1,000 for smaller scale research and pilot programs to over one million targeted for large‐scale, government‐implemented programs (i.e., Madagascar and India).

**Figure 2:**
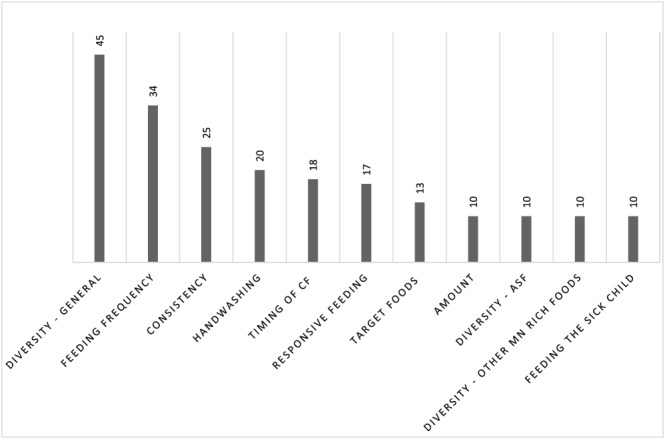
Complementary feeding outcomes. Number of times specific complementary feeding practices were assessed or targeted by the 64 interventions included in this scoping review

Figure 2 presents the complementary feeding practices targeted by included interventions. Notably, dietary diversity was the most common complementary feeding behaviour targeted with 45 interventions focusing more generally on dietary diversity and 33 more specifically on animal source foods, iron‐rich or vitamin A foods, fruits, vegetables, or specific target foods (i.e., eggs and orange flesh sweet potato). Feeding frequency was the next most common outcome (*n* = 34). Amount served at meals (*n* = 10), feeding the sick child (*n* = 10), and using a separate bowl (*n* = 2) were the least common practices emphasized. Forty‐one interventions clearly specified conducting formative research but only 15 explicitly identified the behavioural theory(s) underlying their approach. Theories used included the theory of planned behaviour (*n* = 1; Monterrosa et al., 2013), transtheoretical/stages of change model (*n* = 1; Parvanta et al., 2007), social cognitive/learning theory (*n* = 4; Aboud & Akhter, 2011; Aboud et al., 2008; Aboud et al., 2009; Dickey et al., 2002), socioecological model (Aubel et al., 2004; Kim et al., 2015; Mukuria et al., 2016; Webb Girard et al., 2017), health belief model (*n* = 1; Mulualem et al., 2016), the integrated behaviour model (*n* = 1; Fernald et al., 2016), and adult education theory (Aubel et al., 2004; Webb Girard et al., 2017). Only four studies identified systematic intervention design approaches including Behaviour Centred Design (S. White et al., 2016), the Positive Deviance Approach (Dickey et al., 2002), the Precede–Proceed model (Parvanta et al., 2007), and the Grandmother Project's Change through Culture approach (Aubel et al., 2004; Webb Girard et al., 2017).

Of the five possible platforms for SBC delivery, all but one (Sarrassat et al., 2015) used interpersonal counselling (IPC) strategies whether individual IPC only (*n* = 16), group IPC without individualized IPC (*n* = 18), or a combination of both (*N* = 27). Eighteen reported using media such as radio spots, television, publicly displayed print materials, and radio/video listening groups, all of which were delivered in conjunction with either individual or group IPC or both with the exception of one (Sarrassat et al., 2015) that used media only. Only five reported using policy advocacy/enforcement.

### 3.2 Commonly reported BCTs

Of 96 possible BCTs, the 64 unique interventions included in this review applied a total of 28 unique BCTs. Interventions used a median of six techniques, with the maximum number of techniques used by any one intervention being 13 (Cunningham et al., 2017; Mukuria et al., 2016; Save the Children, 2016) and the minimum being two. All interventions applied BCT 4.1, instruction on how to perform the behaviour. The other commonly applied BCTs in order of prominence included (Table 1) use of a credible source (BCT 9.1, *n* = 46), demonstration of the behaviour (BCT 6.1, *n* = 35) typically in the form of cooking/feeding/recipe demonstrations, and information about health consequences (5.1, *n* = 30). Forty‐three interventions reported strategies to shift the physical or social environment including restructuring the physical environment through cash transfers, agriculture, or kitchen inputs (BCT 12.1, *n* = 19); adding objects to the environment (BCT 12.5, *n* = 29); or restructuring the social environment (BCT 12.2, *n* = 21). The latter was typically through women's empowerment, community mobilization, or engaging with fathers, grandmothers, or other groups that are not typically included in nutrition programming. Few interventions reported provision of households with prompts or cues (BCT 7.1, *n* = 6), social comparison approaches (BCT 6.2, *n* = 12), action planning (BCT 1.2, *n* = 1), goal setting (BCT 1.1, *n* = 4), or problem solving (BCT 1.2, *n* = 12). However, it should be noted that 14 interventions provided informational material during interactions with the families, for example, brochures or recipe cards. Similarly, very few interventions reported providing families with feedback on behaviours as part of the behaviour change strategy (BCT 2.2, *n* = 9), and only one reported using strategies to support families to self‐monitor behaviours (BCT 2.3).

**Table 1:** Commonly applied behaviour change techniques in complementary feeding behaviour change interventions in LMIC

3.1 Social support unspecified (*n* = 25). Often as mother groups or through the care group model; less often through grandmother or fathers groups, neighbour groups, farmer groups; other self‐help groups also mentioned	4.1 Instructions on how to perform behaviours (*n* = 64). Typically frontline health workers, peer mothers, nurses, and physicians providing information about how to perform behaviour. Also, radio/TV spot providing information about how to perform the behaviour; community workshops/skits about how to practice behaviours	5.1 Information on consequences (*n* = 30). Frontline health workers, peer mothers, nurses, and physicians providing information about the benefits of the behaviour for child's health; radio/TV spot providing information about benefits of behaviour/consequences of practicing unsafe behaviours; community workshops/skits about benefits of behaviour/consequences of not practicing recommended behaviours
6.1 Demonstrating the behaviour (*n* = 35). Often in the form of cooking demonstrations, especially of specific foods for diet diversity, enriched, or thick porridge. Typically conducted as part of mother groups or community events. Sometimes used for demonstration of responsive feeding practices	9.1 Credible source (*n* = 46). Use of a respected community member to provide information or encourage behaviour change. Most often, this was a trained community health frontline worker; some interventions used physicians, elected community leaders, or religious leaders	12.2 Restructuring the social environment (*n* = 21). Engagement of influencers (i.e., community leaders, fathers, mother‐in‐laws/elder women) to shift gender, social or cultural norms that hinder optimal IYCF; includes women's education/literacy and empowerment activities
12.5 Adding objects to the environment (*n* = 29). Food/micronutrient supplements, food rations, or special foods (CSB, food products) or agricultural inputs		

*Note*. BCTs included in this table were identified in >20 interventions.

Abbreviations: BCT, behaviour change technique; LMIC, low‐/middle‐income countries.

### 3.3 Estimating effectiveness ratios

We next sought to assess whether patterns emerged between the type and/or quantity of BCT used and intervention effectiveness. Of the 64 unique interventions identified, six (Alive & Thrive India, n.d.; Fernald et al., 2016; Gelli, Becquey, et al., 2017; Gelli, Margolies, et al., 2017; Kadiyala et al., 2016; Kulwa et al., 2014) were in progress and had not reported evaluation data as of August 2018. Forty‐two were randomized controlled or quasi‐experimental studies, and one was a propensity score matched impact evaluation; the remaining interventions applied evaluation strategies that consisted of pre/post designs with no control group (*n* = 15), cross‐sectional surveys (*n* = 1), or were missing baseline data (*n* = 1) and were excluded from these analyses. Of the 42 with adequate evaluation strategies, 31 had data on at least one of five WHO‐defined indicators for optimal complementary feeding indicators (diet diversity, meal frequency, and thick porridge) or macronutrient/micronutrient intakes. Those not reporting on these indicators were excluded from further analysis; they reported on anthropometry (*n* = 5), responsive feeding (*n* = 1), offering or consumption of specific foods (*n* = 3), used summative indices for complementary feeding (*n* = 2), or did not specify the complementary feeding indicator with sufficient detail to classify (*n* = 1). Tables 2 and 3 present the results from these analyses and depict those BCTs applied across interventions by complementary feeding outcome indicator. In general, there was insufficient data to qualitatively surmise associations with specific outcomes as many BCTs had fewer than five applications per outcome. When we collated data across outcomes and examined ratios for the seven BCTs with more than 20 instances of use (Table 1), we note that four had ratios >0.80 (BCTs). These included general social support (BCT 3.1; effectiveness ratios [ER] = 0.96), providing information about the positive or negative health consequences of the behaviour (BCT 5.1, ER = 0.86), demonstration of the behaviour (BCT6.1; ER = 0.88), and adding objects to the environment such as food, supplements, or agricultural inputs (BCT 12.5; ER = 0.85). Less commonly used BCTS with ER > 0.80 included goal setting (BCT 1.1, ER = 1.0), training/support with problem solving (BCT 1.2; ER = 0.9), providing feedback on the behaviour (BCT 2.2; ER = 0.90), providing opportunities to practice/rehearse the behaviour (BCT 8.1; ER = 0.93), and identification of self as a role model (BCT 15.1; ER = 1.0).

**Table 2:** Studies included in behaviour change technique effectiveness ratio estimation

Intervention information		CF behavioural outcomes						SBC approach		
Program name and/or evaluation citations	Country	Thick foods	Diet diversity score	MDD	MAD	Meal frequency	Nutrient intakes	Number BCTs used	BCTs used	Platforms
Alive & Thrive Bangladesh (Menon et al., 2016)	Bangladesh	NA	NA	1	1	1	NA	9	1.2, 3.1, 3.2, 4.1, 5.1, 6.1 9.1 12.2, and 12.5	P/A; IPC‐grp; IPC‐I; CM; media
Alive & Thrive: Vietnam (Rawat et al., 2017)	Vietnam	NA	0	1	1	0	NA	8	3.2, 4.1, 5.1, 6.2, 9.1, 12.1, 12.2, and 13.2	P/A; IPC‐grp; IPC‐I; media
Alive and Thrive: Ethiopia (2018; Kim et al., 2016)	Ethiopia	NA	NA	1	1	1	NA	10	1.1, 1.2, 2.3, 3.1, 4.1, 5.1, 6.1, 9.1, 10.4, and 12.2	P/A; IPC‐grp; IPC‐I; CM; media
(Bhandari et al., 2004)	India	NA	NA	NA	NA	NA	1	7	1.2, 3.1, 4.1, 6.1, 8.1, 9.1, and 12.2	IPC‐grp; IPC‐I; media
(Bhandari et al., 2001)	India	NA	NA	NA	NA	NA	1	9	1.2, 1.4, 2.1, 2.2, 4.1, 6.1, 8.2, 9.1, and 12.5	IPC‐I
(Brasington et al., 2016)	Egypt	NA	NA	0	NA	NA	NA	6	2.7, 3.3, 4.1, 6.1, 9.1, and 12.2	IPC‐I; IPC‐grp
(Crossman et al., 2017)	Kenya	NA	NA	NA	NA	0	NA	3	4.1, 7.1, and 12.2	IPC‐I
Save the Children—Positive Deviance Program (Mackintosh, Marsh, & Schroeder, 2002)	Vietnam	NA	NA	NA	NA	1	NA	8	2.7, 3.1, 4.1, 5.1, 6.2, 8.1, 8.3, and 15.1	IPC‐grp;
NEEP Malawi (Gelli et al., 2017; Gelli & Roschnik, 2017)	Malawi	NA	1	NA	NA	NA	1	6	4.1,6.1, 8.1, 9.1, 12.1, and 12.5	IPC‐grp; IPC‐I
(Kilaru et al., 2005)	India	NA	NA	1	1	1	NA	5	1.2, 2.2, 2.7, 3.1, and 4.1	IPC‐I
(Hoddinott, Ahmed, Ahmed, & Roy, 2017b; Hoddinott, Ahmed, Karachiwalla, & Roy, 2018)	Bangladesh	NA	NA	1	1	1	NA	9	2.2, 3.1, 4.1, 5.1, 6.1, 8.1, 9.1, 12.2, and 12.5	IPC‐grp; IPC‐I
Mama SASHA (Cole et al., 2016; Amy Webb Girard et al., 2017)	Kenya	NA	1	1	NA	0	1	3	4.1, 5.1, and 9.1	IPC‐grp; IPC‐I;
Mamanieva (Mui, 2015; Webb Girard et al., 2016)	Sierra Leone	1	1	1	1	1	NA	8	3.3, 4.1, 5.1, 6.1, 6.2, 8.1, 12.2, and 13.1	IPC‐grp; CM
(Monterrosa et al., 2013)	Mexico	0	NA	NA	NA	NA	1	1	4.1, 5.1, and 9.1	IPC‐I; media
(Mukuria et al., 2016)	Kenya	NA	NA	1	0	1	NA	12	1.2, 3.2, 3.3, 4.1, 6.1, 6.2, 8.1, 9.1, 12.1, 12.2, 12.5, and 13.2	IPC‐grp; IPC‐I; media
(Nikiema et al., 2017)	Burkina Faso	NA	NA	1	NA	1	NA	5	1.2, 2.7, 3.1, 4.1, and 9.1	IPC‐I
HKI HFP Nepal (Osei et al., 2017)	Nepal	NA	NA	1	1	1	NA	6	2.2, 4.1, 6.1, 9.1, 12.1, and 12.5	IPC‐grp; IPC‐I
CARE, Window of Opportunity Program (Owais et al., 2017)	Bangladesh	NA	NA	NA	1	NA	NA	4	3.1, 4.1, 9.1, and 12.2	IPC‐grp; IPC‐I
(Penny et al., 2005)	India	1	NA	NA	NA	NA	1	4	2.7, 4.1, 6.1, and 9.1	IPC‐grp
RAIN (Harris et al., 2016; Kumar et al., 2018)	Zambia	NA	NA	0	1	1	NA	5	4.1, 6.1, 12.1, 12.2, and 12.5	IPC‐grp; IPC‐I; media
(Reinbott et al., 2016)	Cambodia	NA	1	NA	NA	NA	NA	4	3.1, 4.1, 9.1, and 12.2	IPC‐grp; CM
(Roy et al., 2005)	Bangladesh	NA	NA	NA	NA	1	NA	6	2.7, 4.1, 5.1, 6.1, 9.1, and 12.5	IPC‐grp
(Roy et al., 2007)	Bangladesh	NA	NA	NA	NA	1	NA	5	1.2, 3.1, 4.1, 5.1, 6.1, 9.1, and 12.2	IPC‐grp
(Santos et al., 2001)	Brazil	NA	NA	NA	NA	NA	0	3	4.1, 7.1, and 9.1	IPC‐I
Integrated Nutrition and Health Program II (Singh et al., 2017)	India	NA	NA	0	1	1	NA	2	4.1 and 9.1	IPC (unclear grp or I)
Tubaramure (Leroy et al., 2016; Leroy, Olney, & Ruel, 2018)	Burundi	NA	1	NA	NA	1	NA	7	2.7, 4.1, 5.1, 6.1, 9.1, 12.1, and 12.5	IPC‐grp; IPC‐I
(Vazir et al., 2013)	India	NA	NA	NA	NA	NA	1	3	4.1, 6.1, and 12.5	IPC‐I
(S. White et al., 2016)	Indonesia	NA	1	NA	NA	NA	NA	12	1.1, 1.5, 3.1, 3.3, 4.1, 6.1, 6.2, 6.3, 7.1, 9.1, 10.4, and 12.2	IPC‐grp; IPC‐I; media
(Y. Zhang et al., 2016)	China	NA	NA	1	0	1	NA	7	1.2, 2.2, 3.1, 4.1, 6.1, 9.1, and 12.2	IPC‐I

*Note*. 1 = statistically significant positive change in outcome at *P* < .05; 0 = no statistically significant change; NA = outcome not assessed. There were no statistically significant negative changes observed in any of the studies included in effectiveness ratio estimation.

Abbreviations: BCT, behaviour change technique; MDD, minimum diet diversity; MAD, minimum acceptable diet; SBC, social and behaviour change.

**Table 3:** Effectiveness ratios for BCTs used in interventions, presented by complementary feeding indicator and overall

(BCT code) Behaviour change technique	Thick foods	DDS/MDD	Meal frequency	MAD	Nutrient intakes	Ratio
1.1 Goal setting (behaviour)		3/3	2/2	2/2		7/7 (1.00)
1.2 Problem solving		6/6	7/7	3/5	2/2	18/20 (0.90)
1.4 Action planning					1/1	1/1 (1.00)
1.5 Review behavioural goals		1/1				1/1 (1.00)
2.1 Monitoring of behaviours by others without feedback					1/1	1/1 (1.00)
2.2 Feedback on behaviour		4/4	4/4	2/3		10/11 (0.90)
2.3 Self‐monitoring of behaviour/goals		1/1	1/1	1/1		3/3 (1.00)
2.7 Feedback on outcomes of behaviour	1/1	1/4	4/4	1/1	2/2	9/12 (0.75)
3.1 Social support (unspecified)		7/7	7/7	5/6	1/1	20/21 (0.95)
3.2 Social support (practical)		3/5	2/3	2/3		7/11 (0.63)
3.3 Social support (emotional)	1/1	4/5	2/2	1/2		8/10 (0.80)
4.1 Instruction on how to perform the behaviour	2/3	17/22	12/15	12/13	6/7	48/60 (0.80)
5.1 Information about health consequences	1/2	9/10	8/10	5/5	1/1	24/28 (0.86)
6.1 Demonstration of the behaviour	2/2	13/15	11/12	6/8	6/6	38/43 (0.88)
6.2 Social comparison	1/1	5/6	3/4	2/3		11/15 (0.73)
6.3 Information about others' approval		1/1				(1/1) 1.00
7.1 Prompts/cues		2/2	1/2	0/1	0/1	(3/6) 0.50
8.1 Behavioural practice/rehearsal	1/1	5/5	4/4	2/3	2/2	(14/15) 0.93
8.2 Behaviour substitution					1/1	(1/1) 1.00
8.3 Habit formation			1/1			(1/1) 1.00
9.1 Credible source	1/2	14/18	12/15	7/9	5/6	(39/49) 0.79
10.4 Social reward		2/2	1/1	1/1		(4/4) 1.00
12.1 Restructuring the physical environment		7/9	4/6	3/4	2/2	(16/21) 0.76
12.2 Restructuring the social environment	1/2	10/13	5/7	4/5	2/2	(22/29) 0.76
12.5 Adding objects to the environment		8/9	6/7	4/5	4/4	(22/25) 0.85
13.1 Identification of self as role model	1/1	2/2	1/1	1/1		(5/5) 1.00
13.2. Framing/reframing		2/3	1/2	1/2		(4/7) 0.57
15.1 Verbal persuasion about capability			1/1			(1/1) 1.00

*Note*. Data are presented as ratio of effective studies using BCT/all studies using BCT (Martin et al., 2013). BCTS with ratios >0.80 are highlighted.

Abbreviation: BCT, behaviour change technique; DDS, diet diversity score; MDD, minimum diet diversity; MAD, minimum adequate diet.

## 4 DISCUSSION

Several scoping exercises have explored whether complementary feeding education and promotion can achieve behaviour change (Graziose et al., 2018; Lamstein et al., 2014). Despite small and generally positive findings, previous reviews tended to focus on superficial documentation of intervention platforms (community, home, and facility) or were limited to categories of SBC (i.e., interpersonal communication, mass media, and community mobilization) without deeper exploration of what specific techniques were employed across these platforms. Our systematic coding of BCTs using a previously validated taxonomy and assessment of associations with effectiveness found that in general, CF interventions in LMIC have used only one third of the 96 available BCTs. Interventions overwhelmingly depended on strategies that provide instruction, use a credible source to provide instruction, create opportunities for social support, and demonstration of the behaviour. ER for these commonly applied approaches ranged from 0.80 for providing instruction (BCT 4.1) to 0.95 for creating social support opportunities (BCT 3.1), which speaks to the need for combination of strategies.

When mapping the most commonly used BCTs to intervention functions specified in the Behaviour Change Wheel framework (Michie et al., 2014), complementary feeding interventions in LMIC depend predominantly on two of nine intervention functions, namely, education to increase knowledge/understanding (BCTs 4.1, 5.1, 6.1, and 9.1). Although many interventions specified the use of demonstrations (BCT 5.1), it is likely that these techniques were more educational in nature than skill‐building as they did not report providing opportunities for participants to practice learned skills or receive feedback on their progress with learning the skill. Enablement strategies including enhanced social support (BCT 3.1), adding items to the environment (BCT 12.5), or restructuring the social or physical environment (BCT 12.1) were reported; however, they were less frequently reported than education focused approaches. In their mapping of the BCTs used in effective paediatric obesity prevention and treatment, Martin et al. (2013) noted that generalization of the target behaviour (BCT 8.6), opportunities to practice the target behaviour (BCT 8.1), modelling or demonstrating the behaviour (BCT 6.1), and self‐monitoring of behaviours (BCT 2.3) were among the highest scoring BCTs for effective interventions. Similarly, BCTs used in effective obesity management interventions that may be relevant for complementary feeding strategies included environmental restructuring (BCT 12.1), setting and reviewing goals (BCT 1.1, 1.3), use of role models/social comparison (BCT 6.2), social support (BCTs 3.1–3.3), and self‐monitoring of behaviour (BCT 2.4). With the exception of social support, which was included in more than 30 interventions included in this review, complementary feeding interventions rarely included these other BCTs. Only 13 of included interventions reported providing opportunities for caregivers to practice the target behaviours in a supportive environment, eight indicated goal setting and/or monitoring of goals, and only one provided opportunities or supports for caregivers or families to monitor infant feeding practices themselves. Rather, behavioural monitoring was performed by a trained health worker, peer leader, or other monitor; it was not always clear in publications whether those monitoring caregivers' behaviour provided feedback on the monitored behaviour. Similarly, no interventions reported the use of generalization, the transfer of skills learned in one setting, or circumstance to other circumstances. For example, if mothers are taught how to use orange flesh sweet potato in a specific recipe in place of Irish or white sweet potato, then generalization would involve mothers identifying other recipes or times to use the orange sweet potato, not only in the recipe they were taught.

Persistent undernutrition juxtaposed against the growing prevalence of child overnutrition highlights how families in both high and low resource contexts struggle to optimally feed their young children. Although we recognize the need for context‐specific approaches, it is likely there are valuable lessons to be learned from sharing of effective behaviour change strategies between those working to prevent young child overweight and obesity in high‐income countries and those working to improve infant feeding in LMIC. Platforms that promote the valuing and sharing of experiences and lessons learned between these two, often siloed groups, are needed to optimize child diets and growth in all contexts.

We should note that in this systematic review, interventions typically compared the impacts of a multicomponent package against receipt of against an existing standard of care, for example, infant and young child feeding counselling provided by health care workers as part of routine clinic visits or nothing at all. Given that many of the interventions had multiple components and used a mix of delivery platforms, it is unclear which intervention components contributed to behaviour change. Lack of clarity regarding the relative effectiveness of specific intervention components precludes identifying the most optimal package in a given context—one that is efficient, cost‐effective, and scalable. Additional research is needed to test intervention components against one another to identify combinations of components in a given context that produce the most optimized package. One promising approach in the field of behavioural intervention research that enables such testing is the Multiphase Optimization Strategy (Collins, Murphy, Nair, & Strecher, 2005). This approach has been applied, for example, in the fields of HIV (Collins, Kugler, & Gwadz, 2016; Gwadz et al., 2017), smoking cessation (Baker et al., 2016; Collins et al., 2011), and to develop optimized behavioural interventions.

### 4.1 Limitations

First, because the search strategies for previous systematic reviews of complementary feeding interventions aligned with the goals of our own review, we did not reconstruct and conduct an original search of the literature. The exception was in the updating of the review to include studies published since December 2016. The identification and mapping of BCTs was not limited to only those interventions with evaluation data. As such, we thoroughly reviewed studies both included and excluded from these previous reviews to ensure we were not excluding relevant interventions that were excluded from other reviews based on evaluation design. In some cases, excluded interventions were listed in the original review documentation and, in other cases, we contacted review authors for lists of excluded studies to review. We recognized that our strategy may have resulted in the exclusion of some relevant studies. However, given the comprehensive nature of the original reviews' search strategies, our review of both included and excluded interventions and additional snowball searching for intervention details and of the grey literature, we are confident that the number of relevant interventions excluded from this scoping review is minimal.

A second limitation to our work was the stringent coding strategy we applied, per the protocol outlined by the BCT taxonomy training. This protocol entailed mapping only those BCTs with sufficient detail to adequately and correctly code. In many cases, intervention details were insufficiently described in peer‐reviewed manuscripts and publically available reports. Despite additional snowball searching, we were unable to locate details for approximately half of the interventions included in this review. As such, we likely underreport BCTs, having coded as absent those techniques that intervention designers would argue were present but were not reported. For example, 45 interventions used group education. One could assume that group‐based education could be designed and implemented to enable social support for a given set of practices; however, many of these interventions lacked sufficient detail to all.

This lack of reporting detail has emerged as problematic for other health behaviours, including exclusive breastfeeding (Gosselin, 2016). The ability to reproduce interventions is critical not only for identifying what works and does not work but also to enable scale up of strategies that do work. Thus, it is imperative that publications and reports about behaviour change interventions are sufficiently detailed to include not only the platforms and general approaches but also underlying behavioural theories, intervention design frameworks, program theory of change, and BCTs. Only when this level of detail is provided will interventions be reproducible and verifiable. Comprehensive guidelines exist for reporting behaviour change interventions (Abraham, Johnson, de Bruin, & Luszczynska, 2014; Albrecht, Archibald, Arseneau, & Scott, 2013; Johnston, 2014) including a checklist for reporting on group behaviour change interventions. However, greater enforcement of these guidelines is needed by both donors/funders of interventions and publishers. As well, given the small number of studies for many of the BCTs, we argue there is a need for rigorously designed and documented behaviour change research in the field of complementary feeding to identify those techniques that contribute to the initiation and maintenance of behaviour change not only over time but also from one child to the next.

### 4.2 Conclusions

In their review of behaviour change interventions for infant feeding in LMIC, Briscoe and Aboud noted “behaviours may be difficult to change because they are habitual, normative and preventive. Habitual behaviours are difficult to change because they are performed automatically without much thought; normative behaviours bear the weight of tradition and approval; and preventive behaviours often lack a salient immediate outcome” (Briscoe & Aboud, 2012, p. 590). Global reviews of progress on infant feeding practices reflect these challenges (Lutter et al., 2011; J. M. White et al., 2017). As Atkins and Michie note, diet change programs have typically taken an “ISLAGIATT—It seemed like a good idea at the time‐approach” to intervention design (Atkins & Michie, 2015). Others have similarly reflected on the lack of theoretical foundations, formative research, theory of change, or systematic intervention design frameworks used in the development of SBC strategies for infant feeding (Graziose et al., 2018; Iannotti et al., 2017; Lutter et al., 2011; Pelto et al., 2016). Given the slow progress to date on improving infant feeding practices, coupled with current global goals to reduce both stunting and child overweight, the lack of reporting for interventions and the use of unsystematic, atheoretical, or nonevidence‐based approaches to design complementary feeding behaviour change interventions could be viewed as inefficient at best and unethical at worst. Our findings speak to the urgent need for greater public sharing of intervention details among the global IYCF community—inclusive of both high and LMIC—especially related to design, underlying theory of change, implementation, and lessons learned. Such public sharing is critical for the identification, verification, replication, and scale up of effective change strategies.

## CONFLICTS OF INTEREST

The authors declare they have no conflicts of interest.

## CONTRIBUTIONS

AWG and LG conceptualized the research question and analytical approach. EW and SS abstracted all data and mapped behaviour change techniques. AWG wrote the first and subsequent drafts of the article. All authors contributed to critically revising the article and gave final approval of the version to be published.

## Supporting information


**Figure S1.** Behaviour Change Techniques Taxonomy of 93 hierarchically‐clustered techniques; adapted from Michie et al., 2013 Ann Behav Med 46(1), 81‐95 doi:10.1007/s12160‐013‐9486‐6
